# Diagnostic Value of miR-21, miR-122, miR-145, and miR-146a in Acute Pancreatitis: Findings from the PASEVO Cohort in Southeastern Romania

**DOI:** 10.3390/ijms27052264

**Published:** 2026-02-27

**Authors:** Diana Iosif, Costel Stelian Brînzan, Andra Iulia Suceveanu, Iulia Cîndea, Viorel Gherghina, Marius Dragoș Prăzaru, Alina Doina Nicoară, Felix Voinea, Miruna-Gabriela Vizireanu, Adrian Neluțu Mitroi, Anca Florentina Mitroi, Adrian Paul Suceveanu

**Affiliations:** 1 Doctoral School of Medicine, Ovidius University of Constanta, 900470 Constanta, Romania; dianaiosif297@gmail.com (D.I.); marius.prazaru@365.univ-ovidius.ro (M.D.P.); 2 Department of Intensive Care Unit, St Andrew Apostle Emergency Hospital, 900591 Constanta, Romania; iulia.candea@365.univ-ovidius.ro (I.C.); viorel.gherghina@365.univ-ovidius.ro (V.G.); 3 Center for Research and Development of the Morphological and Genetic Studies of Malignant Pathology (CEDMOG), “Ovidius” University, 900591 Constanta, Romania; miruna.vizireanu@365.univ-ovidius.ro (M.-G.V.); adrian.mitroi@365.univ-ovidius.ro (A.N.M.); anca.mitroi@365.univ-ovidius.ro (A.F.M.); 4 Faculty of Medicine, Ovidius University of Constanta, 900470 Constanta, Romania; alina.nicoara@365.univ-ovidius.ro (A.D.N.); felix.voinea@365.univ-ovidius.ro (F.V.); adrian.suceveanu@365.univ-ovidius.ro (A.P.S.); 5 Department of Gastroenterology, St Andrew Apostle Emergency Hospital, 900591 Constanta, Romania; 6 CFR Clinical Hospital Constanta, 900123 Constanta, Romania

**Keywords:** acute pancreatitis, severe acute pancreatitis, microRNA, serum biomarkers, miR-21, miR-122, miR-145, miR-146a, ROC analysis

## Abstract

Acute pancreatitis (AP) is an inflammatory condition of the pancreas that initiates a cascade of inflammation, which can cause pancreatic damage or spread to other organs. The severity of AP ranges from mild acute pancreatitis (MAP) to severe acute pancreatitis (SAP). While standard diagnostic tools include contrast-enhanced CT scans, clinical scoring systems like APACHE II and Ranson, and laboratory tests, miRNAs have emerged as crucial regulators of gene expression involved in various physiological and pathological processes. Over the last decade, research has demonstrated that significant alterations in miRNA levels are integral to AP development. Since one miRNA can target multiple genes, they serve as key modulators of disease progression.

## 1. Introduction

AP is a sudden inflammatory disorder of the pancreas that can spread to surrounding tissues and, in some cases, cause damage to distant organs, with an annual incidence of 13 to 45 cases per 100,000 people [1,2]. The disease arises from a variety of causes. Still, the triggering event is usually the unintentional activation of digestive enzymes within the gland, setting off a cascade of inflammation that can be limited to the pancreas or can lead to multiple-organ dysfunction. Factors commonly implicated include trauma to the pancreatic duct, gallstones, retrograde bile flow, alcohol consumption, dyslipidemia, complications following endoscopic retrograde cholangiopancreatography (ERCP), certain drug reactions, and heritable mutations [3,4].

AP is characterized by variable clinical severity. While some patients experience mild, self-limiting episodes MAP, approximately 10–20% develop a fulminant, life-threatening condition known as SAP, which is associated with a high mortality rate of 20% [5]. The exact mechanisms by which different etiological factors trigger AP are not fully understood. However, it is widely accepted that the early events in AP originate in the acinar cells of the pancreas [6]. Initial injury to these cells initiates a local inflammatory response. If this response becomes amplified, it can progress to systemic inflammatory response syndrome (SIRS) [7]. SIRS, in turn, can cause remote organ injury, ultimately leading to multiple organ dysfunction syndrome (MODS). MODS is the leading cause of both morbidity and mortality in patients with severe AP [8]. Therefore, understanding the underlying molecular mechanisms of SAP is of urgent clinical importance.

Among the molecular regulators currently under investigation, microRNAs (miRNAs), a class of small, evolutionarily conserved, endogenous non-coding RNAs, have emerged as key players [9]. Typically, 19 to 25 nucleotides in length, these molecules naturally occur within cells and regulate gene expression at the post-transcriptional level [10,11]. MiRNAs are now recognized for their involvement in a wide range of physiological and pathological processes [12]. Functional studies have demonstrated their roles in essential cellular functions, including apoptosis, proliferation, and differentiation [13,14,15]. Notably, a single miRNA can target multiple genes, establishing them as critical modulators of cellular homeostasis and disease pathogenesis [16].

In 2008, miRNAs were also discovered in human blood, where they have been detected in plasma, platelets, erythrocytes, and nucleated blood cells [17,18]. Circulating miRNAs, particularly those found in plasma, exhibit remarkable stability. They can endure harsh conditions such as extreme pH changes, prolonged storage at room temperature, and multiple freeze–thaw cycles [19].

This intrinsic resilience makes them an up-and-coming class of emerging biomarkers, due to their detectability in bodily fluids, tissue-specific expression profiles, and robust stability. MiRNAs exert their regulatory function by silencing a broad range of messenger RNA (mRNA) targets, thereby influencing a wide array of biological processes [20]. Although many studies support the diagnostic and prognostic value of miRNAs in both tissue and blood samples from AP patients, the findings often exhibit substantial variability and, in some cases, conflicting results regarding specific miRNAs [21,22,23]. While previous research has clarified several molecular roles of these miRNAs—such as miR-146a’s anti-inflammatory effect via TRAF6-NF-κB [24], miR-145’s link to oxidative stress and apoptosis [25], and miR-122’s role in aggravating disease by impairing intestinal barrier function [26]—most evidence remains experimental. Bioinformatic studies have also identified miRNA-regulated pathways in metabolic pancreatitis [27], while miR-21 has been proposed for risk stratification [28]. However, as current data largely derive from animal models or computational analyses, clinical validation in human cohorts remains essential to confirm their utility as biomarkers.

Our objective was to assess the clinical utility of four circulating mature miRNAs—hsa-miR-21, hsa-miR-122, hsa-miR-146a, and hsa-miR-145 as potential biomarkers for distinguishing patients with AP from healthy controls and for differentiating between MAP and SAP. The analysis was conducted on 50 serum samples obtained from AP patients in the southeastern region of Romania. These miRNAs were selected based on prior evidence of dysregulation in inflammatory diseases, alongside their biological relevance to pancreatic injury pathways.

## 2. Results

### 2.1. The Demographic and Clinical Characteristics of Participants

A total of 50 patients with a confirmed diagnosis of AP, 23 with MAP and 27 SAP, alongside 24 healthy control subjects, were included in the analysis. The demographic and clinical characteristics of all study groups are summarized in Table 1. No significant differences were observed in age, sex, or geographical distribution between AP patients and the control group (*p* > 0.05). Compared with patients with MAP, those with SAP exhibited significantly higher serum amylase and CRP levels, as well as elevated APACHE II and Ranson scores (*p* < 0.05), consistent with increased disease severity. In addition, a significant difference in geographical distribution was observed between the MAP and SAP subgroups (*p* < 0.05). Moreover, the etiological distribution of PA and multi-organ dysfunction were also analysed. Biliary pancreatitis was observed in 8 MAP and 13 SAP patients. Alcohol-related pancreatitis occurred in 6 MAP and 7 SAP patients. Metabolic pancreatitis was identified in 2 MAP and 6 SAP patients, while post-interventional pancreatitis was observed in 5 MAP and 2 SAP patients. Neurological dysfunction was present in 20 MAP and 12 SAP patients. Both cardiovascular and respiratory dysfunctions were present in 4 MAP and 12 SAP patients, while both renal and hematological dysfunctions were observed in 6 MAP cases and 13 SAP cases. Fluid-coagulation abnormalities occurred in 7 MAP and 16 SAP patients. No statistically significant differences in both etiological distribution and multi-organ dysfunction were found between AP groups (*p* > 0.05).

### 2.2. Expression Levels of the Selected miRNAs in AP Patients

**Figure 1:**
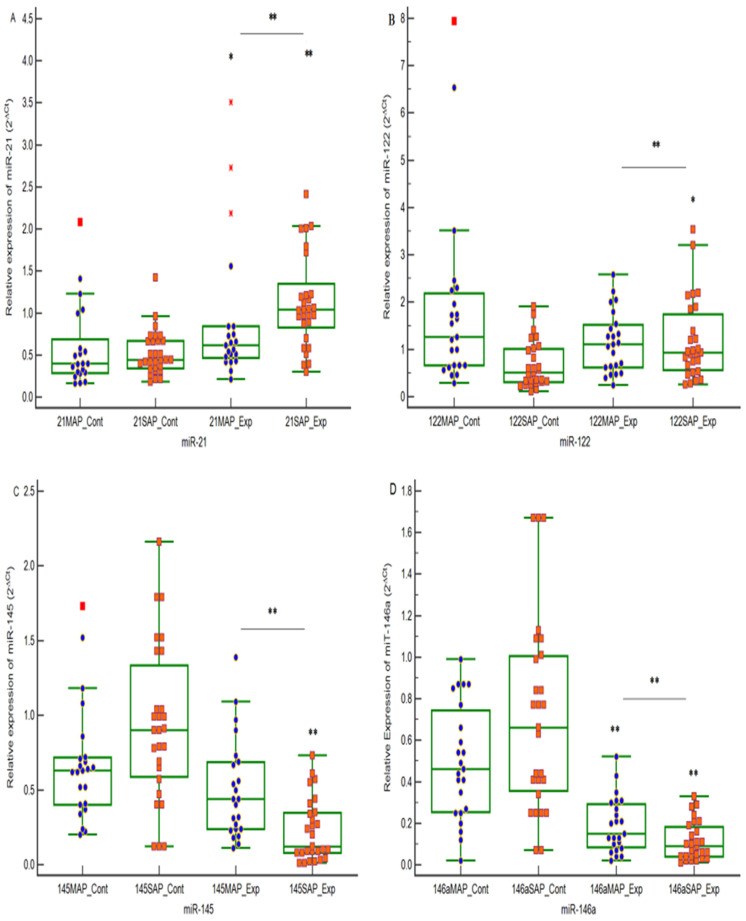
The relative concentrations of miR-21, miR-122, miR-145 and miR-146a in the serum samples from the Control (*n* = 24), MAP (*n* = 23), and SAP (*n* = 27) groups (**A**–**D**). Ct values were converted to relative concentrations, normalized to miR-16 values, and calculated using the comparative Ct method (2^−ΔΔCt^). Each point represents the mean of triplicate samples. * *p* < 0.05; ** *p* < 0.001.

Our findings revealed that all four investigated miRNAs exhibited dysregulated expression patterns in patients with AP. As shown in Figure 1, serum miR-21 levels were significantly higher in both MAP (*p* = 0.04) and SAP (*p* = 0.0003) patients compared with healthy controls. Similarly, serum miR-122 expression was significantly increased in SAP patients (*p* = 0.0105) relative to controls, whereas no significant difference was observed between MAP patients and controls (*p* > 0.05). Furthermore, serum miR-21-5p and miR-122 levels were markedly elevated in SAP compared with MAP patients (both *p* < 0.0001).

Conversely, miR-145 showed significantly lower expression levels in SAP patients (*p* < 0.0001) compared with controls, whereas no significant difference was detected between MAP patients and controls (*p* > 0.05). Likewise, miR-146a expression was reduced in MAP and SAP patients relative to controls (*p* < 0.0001). Moreover, pairwise comparisons revealed that both miR-145 and miR-146a were significantly lower in the SAP group compared with MAP (both *p* < 0.0001).

### 2.3. Correlations Between miRNA Levels and Clinical Variables

We evaluated whether the patients’ clinical features, as well as disease etiology and the presence of organ dysfunctions, were associated with circulating miRNA levels. As shown in Table 2, miR-146a exhibited a strong negative correlation with serum amylase; in contrast, miR-21 showed a strong positive correlation with Balthazar CT grade, Ranson score, APACHE II score and Leukocytosis, and a negative correlation with serum calcium levels. Both geographical distribution and Balthazar CT grade demonstrated strong negative correlations with miR-145. In addition, miR-122 showed a strong positive correlation with Balthazar CT grade, APACHE II score, Ranson score, and all dysfunctions analysed.

### 2.4. ROC Analysis of the Selected Serum miRNAs in AP Patients

**Figure 2:**
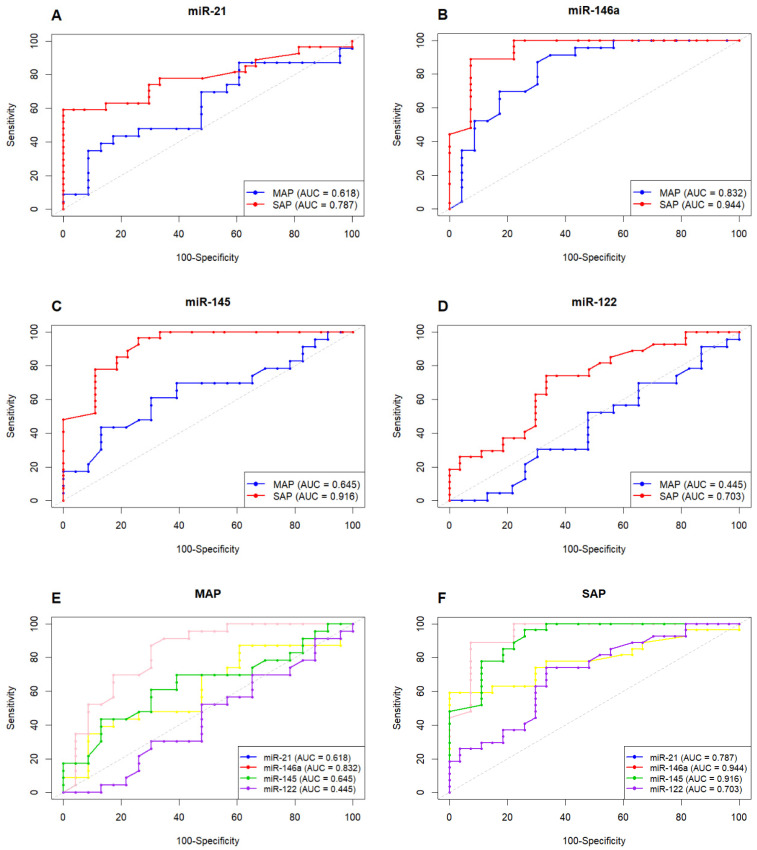
ROC curve analysis of miR-21, miR-146a, miR-145, and miR-122 to assess their diagnostic potential in acute pancreatitis (AP). (**A**) miR-21: AUC = 0.61 in MAP (Sensitivity: 39.13%, Specificity: 86.96%; *p* = 0.16) and AUC = 0.78 in SAP (Sensitivity: 59.26%, Specificity: 100%; *p* < 0.001). (**B**) miR-146a: AUC = 0.83 in MAP (Sensitivity: 86.96%, Specificity: 69.57%; *p* < 0.001) and AUC = 0.94 in SAP (Sensitivity: 88.89%, Specificity: 92.59%; *p* < 0.001). (**C**) miR-145: AUC = 0.64 in MAP (Sensitivity: 60.87%, Specificity: 69.57%; *p* = 0.08) and AUC = 0.91 in SAP (Sensitivity: 96.30%, Specificity: 74.07%; *p* < 0.001). (**D**) miR-122: AUC = 0.44 in MAP (Sensitivity: 95.65%, Specificity: 21.74%; *p* = 0.52) and AUC = 0.70 in SAP (Sensitivity: 74.07%, Specificity: 66.67%; *p* = 0.004). (**E**,**F**) Combined miRNA panel (miR-21, miR-146a, miR-145, and miR-122): AUC = 0.85 in MAP (Sensitivity: 86.96%, Specificity: 78.26%; *p* < 0.001) and AUC = 0.99 in SAP (Sensitivity: 96.30%, Specificity: 100%; *p* < 0.001).

We performed ROC curve analyses to evaluate the diagnostic accuracy of the four selected miRNAs in distinguishing MAP and SAP patients from healthy controls. The ROC curve revealed all four miRNAs were significantly dysregulated in SAP patients (*p* < 0.05), and only miR-146a showed significant diagnostic potential in MAP patients (Figure 2, Table 3).

Among them, miR-146a demonstrated the highest discriminatory power, with an AUC of 0.83 in MAP patients (Sensitivity: 86.96%, Specificity: 69.57%) and 0.94 in SAP patients (Sensitivity: 88.89%, Specificity: 92.59%). miR-21 showed notable diagnostic value only in SAP patients (AUC: 0.78; Sensitivity: 59.36%; Specificity: 100%). miR-145 and miR-122 were also significant only in SAP, with AUCs of 0.91 (Sensitivity: 74.07%, Specificity: 66.67%) and 0.70 (Sensitivity: 74.07%, Specificity: 66.67%), respectively.

To improve diagnostic performance, we developed a combined miRNA panel comprising miR-21, miR-146a, miR-145, and miR-122. The panel achieved an AUC of 0.85 for MAP patients and 0.99 for SAP patients, highlighting the superior diagnostic value of multiplex miRNA profiling (Figure 2, Table 4).

## 3. Discussion

AP is one of the leading causes of gastrointestinal hospitalization worldwide and remains a potentially life-threatening condition [28]. Although most cases are mild and self-limiting, approximately 10–20% of patients develop SAP, which is associated with systemic inflammation, multi-organ failure, and high mortality rates. Early diagnosis and adequate stratification of disease severity are, therefore, crucial for improving clinical outcomes. The current diagnostic standard relies on contrast-enhanced CT and clinical scoring systems such as APACHE II and the Ranson criteria. However, CT imaging is costly and exposes patients to radiation, while scoring systems can be cumbersome and may not be sensitive enough during the early, rapidly evolving stages of disease. These limitations underscore the need for reliable, non-invasive biomarkers capable of detecting AP early and predicting progression to severe disease [5].

Circulating miRNAs should not be interpreted as substitutes for established diagnostic and severity assessment tools, such as contrast-enhanced CT imaging or clinical scoring systems like APACHE II and Ranson. Instead, these molecules should be viewed as complementary or adjunctive biomarkers that enhance the diagnostic framework for SAP. Their principal clinical value stems from their high stability in biofluids and resistance to enzymatic degradation, qualities largely attributed to their encapsulation within exosomes or their association with protein complexes like Argonaute proteins [23]. In the context of AP, several studies have demonstrated altered serum miRNA profiles, suggesting that these molecules reflect both local pancreatic injury and systemic inflammatory responses [29,30]. Consequently, rather than replacing existing diagnostic standards, circulating miRNAs offer a minimally invasive clinical advantage in early risk stratification and the longitudinal monitoring of disease progression.

In SAP, circulating miRNAs are unlikely to reflect only pancreatic injury. The disease is associated with systemic inflammatory response and frequently progresses to multi-organ dysfunction involving the lungs, kidneys, and cardiovascular system. Consequently, circulating miRNA levels probably represent a systemic inflammatory signal rather than isolated pancreatic necrosis, which may explain their correlation with clinical severity scores. Therefore, these miRNAs should be interpreted as markers of the host response to injury rather than pancreas-specific biomarkers.

miR-21 is generally regarded as a pro-fibrotic microRNA, and its upregulation has been associated with pancreatic fibrosis and activation of the NF-κB pathway [31]. miR-122 has been linked primarily to liver injury and lipid metabolism, miR-146a is involved in the modulation of innate immune responses, while miR-145 plays a key role in regulating apoptosis and smooth muscle differentiation [32,33,34].

However, existing studies have yielded inconsistent findings regarding the diagnostic or prognostic utility of these miRNAs in acute pancreatitis, underscoring the need for further validation in well-defined patient cohorts. In this context, the present study investigated the clinical relevance of four circulating mature miRNAs (miR-21, miR-122, miR-145, and miR-146a) in serum samples collected from patients with AP in the southeastern region of Romania.

Our study found that miR-21 levels were significantly elevated in both MAP and SAP patients compared to healthy controls, with a more pronounced increase in SAP patients. This gradual rise with disease severity indicates that miR-21 might play an important role in acute pancreatitis development, possibly by promoting inflammatory activation and enhancing fibrosis in the pancreatic tissue [35]. These findings align with a recent study identifying circulating miR-21 as a key driver of disease progression and a potential tool for the risk stratification of acute pancreatitis [28]. Additionally, miR-21 showed strong positive correlations with Balthazar CT grade, Ranson score, and APACHE II score, and a negative correlation with serum calcium levels, linking it closely to disease severity, systemic inflammation, and pancreatic damage [27]. ROC curve analysis further indicated that miR-21 had significant diagnostic value mainly in SAP patients, implying that while it may not reliably detect early or mild AP, it is a useful biomarker for identifying patients at higher risk of severe disease progression. By demonstrating its clinical performance within a combined panel, our study highlights the advantage of miR-21 in achieving high-precision diagnostic outcomes for the most severe cases.

miR-145 is widely recognized for its role as a tumor suppressor, being consistently downregulated in several types of cancer, including ovarian, cervical, breast, and colorectal malignancies [36]. Beyond its relevance in oncogenesis, miR-145 is also involved in the regulation of non-malignant conditions such as asthma, diabetes, and metabolic disorders, where its altered expression may contribute to disease pathophysiology and hold therapeutic potential [37]. In the present study, we observed a significant downregulation of miR-145 in SAP patients compared with healthy controls, whereas no statistically significant difference was identified between MAP patients and controls. This observation aligns with experimental findings where miR-145 expression was significantly altered in rat models of chronic pancreatitis, showing a direct relationship with oxidative stress, endoplasmic reticulum stress, and apoptosis [25]. While previous studies focused on chronic experimental models, our clinical data extend these findings to the acute setting, demonstrating that miR-145 reduction is specifically linked to SAP and radiological disease severity as indicated by the Balthazar CT grade [38]. ROC curve analysis demonstrated that miR-145 could effectively distinguish SAP patients from healthy individuals. This high discriminative power supports its utility as a potential biomarker for identifying patients at risk of severe disease progression, although its limited performance in MAP suggests it may be more reliable in advanced stages of pancreatitis than for early disease detection.

miR-146a is a well-documented negative regulator of pro-inflammatory signaling, particularly through the NF-κB pathway, where it acts as a feedback inhibitor to restrain excessive cytokine production [39]. Its decreased expression in AP may therefore contribute to the uncontrolled inflammatory response characteristic of severe disease [40]. This is further supported by its inverse correlation with biochemical and clinical severity markers, indicating that diminished miR-146a levels may reflect the failure of endogenous anti-inflammatory mechanisms.

Similarly, miR-146a expression was significantly reduced in both MAP and SAP patients compared with healthy controls, with a more pronounced downregulation observed in SAP. This stepwise decrease in miR-146a levels according to disease severity suggests a potential loss of its regulatory function during the progression of acute pancreatitis. The diagnostic relevance of this miRNA was confirmed by ROC curve analysis, which showed the highest discriminative performance among all individual miRNAs tested. Our clinical findings regarding the significant reduction in miR-146a in both MAP and SAP patients complement the experimental data provided by Yang et al., who demonstrated that miR-146a acts as an anti-inflammatory modulator via the TRAF6-NF-κB signaling pathway [24]. While their study highlights its protective role in mice, our data suggest that lower circulating levels of miR-146a in humans correlate with higher diagnostic accuracy for AP, potentially reflecting a depletion of this protective mechanism during the acute phase.

Furthermore, serum miR-122, a molecule extensively studied in liver diseases for its high liver specificity [41], also appears to play a key role in acute pancreatitis [5]. Our study found elevated serum levels of miR-122 exclusively in SAP patients. This finding is consistent with recent research suggesting that the overexpression of miR-122 aggravates acute pancreatitis by disrupting intestinal epithelial barrier integrity through the downregulation of occludin, thereby facilitating systemic inflammatory responses [27]. In our cohort, this is clinically reflected by the strong positive correlations observed between miR-122 and Balthazar CT grade, APACHE II, Ranson scores, and all organ dysfunctions included in the study, emphasizing its connection with both local injury and systemic complications like organ failure [42]. The positive correlation between expression levels of miR-122 and multi-organ dysfunctions can be explained by the fact that miR-122 reflects secondary hepatic injury and intestinal barrier dysfunction during systemic inflammatory response syndrome [27]. Although its performance is moderate compared to other miRNAs in our panel, its specificity and its role in aggravating disease severity through intestinal barrier impairment suggest that miR-122 is a crucial component of a multi-miRNA diagnostic approach.

Combining miR-21, miR-146a, miR-145, and miR-122 into a panel significantly improved diagnostic accuracy for both MAP and SAP, with higher AUC values than individual markers. These panels also showed excellent sensitivity and specificity, highlighting the benefit of a combined miRNA panel to overcome the limitations of single markers [27]. From a practical perspective, circulating miRNA testing is feasible because it relies on qRT-PCR, a method already available in most hospital molecular laboratories. The purpose of these biomarkers is not population screening but early triage. Identifying patients at risk for severe acute pancreatitis within the first 24–48 h may guide closer monitoring and timely ICU admission. However, standardization of sampling and processing remains necessary before routine clinical implementation.

However, this study has some limitations. First, the relatively small sample size (n = 50) may limit the statistical power and the ability to detect more subtle differences. Due to this constraint, multivariate regression was not performed to avoid potential overfitting and unstable estimates. Larger prospective cohorts are therefore required to validate the independent predictive value of these miRNAs and to integrate them into robust clinical prediction models. Second, the miRNA profiling was performed only on serum samples without extracting exosomal miRNAs, which could influence results since many miRNAs are contained in exosomes [43]. Another limitation is that AP can be associated with systemic complications, including organ failure [44]. Thus, the expression of the selected miRNAs may reflect not only pancreatic pathology but also systemic inflammation or damage to other organs [45,46].

## 4. Materials and Methods

### 4.1. Study Design

The study of serum miRNA expression was conducted on 50 patients admitted with AP to the Intensive Care Unit of the Emergency County Clinical Hospital in Constanța, Romania, between October 2024 and August 2025. The study received approval from the Local Ethics Committee for Clinical and Research Developmental Studies, and written informed consent was obtained from all participants prior to biological sample collection (approval no. 45661/09.08.2023). Biological samples were collected within 48 h of hospital admission to capture the established phase of the systemic inflammatory response syndrome (SIRS). This timeframe was selected because the 24–48 h window aligns with the 2012 Revised Atlanta Classification’s criteria for identifying persistent organ failure (>48 h), which is the hallmark of SAP [47]. While admission samples may reflect transient metabolic shifts or be influenced by initial fluid resuscitation, this interval allows for a more accurate differentiation between transient inflammation and established necrosis or organ dysfunction, especially in ICU patients.

Based on disease severity, patients were categorized into MAP and SAP, with 24 age- and gender-matched healthy individuals serving as controls. Serial laboratory tests were performed throughout hospitalization to monitor biomarker levels.

Consequently, SAP was operationally defined in our cohort by the presence of persistent organ failure and/or local complications, identified by meeting at least one of the following criteria: APACHE II score ≥ 8, Ranson score ≥ 3, Balthazar CT grade D–E, or confirmed local complications. Patients who did not meet these criteria, exhibiting an APACHE II score < 8, Ranson score < 3, and Balthazar CT grade A–C without organ failure, were classified as MAP.

### 4.2. Preparation of Blood Samples

To maintain pre-analytical quality, whole blood from patients was collected in BD Vacutainer^®^ SST™ II Advance tubes, Plymouth, United Kingdom containing a clot activator and processed within one hour of collection. After allowing 20 min for clot formation at room temperature (RT), the tubes were centrifuged at 3000 rpm and 4 °C for 10 min. The upper serum phase was carefully isolated and transferred to a new Eppendorf tube, ensuring the intermediate leukocyte layer remained undisturbed to avoid cellular contamination. All samples underwent visual inspection for hemolysis, discoloration, or contamination. Hemolysis levels were quantitatively assessed using a NanoDrop™One spectrophotometer, Thermo Fisher Scientific, Waltham, MA, USA and affected samples were excluded from downstream analysis. To eliminate residual cellular nucleic acids adhering to debris, serum samples were subjected to a second centrifugation step (3000 rpm, 4 °C, and 15 min). The clarified supernatant was aliquoted into nuclease-free cryotubes, mixed with 50 µL of RNA later^®^ stabilization solution, and stored at −80 °C until further processing.

### 4.3. MicroRNA Isolation from Serum Samples

Total miRNA was extracted from 200 µL of serum using the miRNeasy Serum/Plasma Kit (QIAGEN^®^, Hilden, Germany) following the manufacturer’s protocol. Briefly, serum samples were homogenized with 1 mL of QIAzol^®^ Lysis Reagent and 2 µL of carrier RNA (1 µg/µL). After 5 min of incubation at RT, 200 µL of chloroform was added, followed by vigorous shaking and centrifugation at 15.000 rpm for 15 min at 4 °C. The aqueous phase was transferred to a fresh tube, and ethanol was added to promote RNA binding to the RNeasy MinElute^®^ spin column.

Membrane-based purification was performed via sequential centrifugation steps using buffers RWT and RPE for washing. After drying the column, RNA was eluted with 20 µL of RNase-free water by centrifugation at 15.000 rpm for 1 min. Quantification of each miRNA from the perspective of qualitative, quantitative, and integrity was measured spectrophotometrically with NanoDrop One Spectrophotometer (Thermo Fisher Scientific, Waltham, MA, USA), fluorometric with Qubit 3.0 Fluorometer (Thermo Fisher Scientific), and microcapillary electrophoresis with 2200 TapeStation Bioanalyzer (Agilent Technologies GmbH, Waldbronn, Germany).

### 4.4. Reverse Transcription of miRNA to Complementary cDNA

Selected human miRNAs were reverse-transcribed into cDNA using the TaqMan™ Advanced miRNA cDNA Synthesis Kit (Applied Biosystems, San Diego, CA, USA) according to the manufacturer’s protocol. To summarize the procedure, 2 µL of previously extracted total RNA (ranging from 1 to 10 ng) were combined with 3 µL of Total Poly(A) Reaction Mix (comprising 0.5 µL 10× Poly(A) Buffer, 0.5 µL ATP, 0.3 µL Poly(A) Enzyme, and 1.7 µL of RNase-free water). The mixture was subjected to standard cycling conditions (37 °C for 45 min, 65 °C for 10 min, and then held at 4 °C). Afterward, 10 µL of the Ligation Reaction Mix (comprising 3 µL of 5× DNA Ligase Buffer, 4.5 µL of 50% PEG 8000, 0.6 µL of 25× Ligation Adaptor, 1.5 µL of RNA Ligase, and 0.4 µL of RNase-free water) was added to each tube containing the product of the poly(A) tailing reaction. Following an incubation step in a thermal cycler at 16 °C for 60 min, with a final hold at 4 °C, the reverse transcription (RT) reaction was initiated. This was achieved by adding 15 µL of RT Reaction Mix (comprising 6 µL of 5× RT Buffer, 1.2 µL of dNTP Mix, 1.5 µL of 20× Universal RT Primer, 3 µL of 10× RT Enzyme Mix, and 3.3 µL of RNase-free water) to the product of the adaptor ligation reaction. The RT reaction proceeded under standard cycling conditions (42 °C for 15 min, 85 °C for 5 min, and then held at 4 °C). Finally, for the miR-Amp reaction, 45 µL of the total miR-Amp Reaction Mix (consisting of 25 µL of 2× miR-Amp Master Mix, 2.5 µL of 20× miR-Amp Primer Mix, and 17.5 µL of RNase-free water) were combined with 5 µL of the RT reaction product and incubated as follow: initial activation at 95 °C for 5 min, followed by 14 cycles at 95 °C for 3 s and 60 °C for 30 s. Finally, the reaction was stopped at 99 °C for 10 min and then held at 4 °C.

### 4.5. Measurement of miRNA Levels Using Real-Time Quantitative PCR

The real-time PCR analysis was performed in triplicate for each sample using the ABI 7500 Fast qPCR System. Each reaction consisted of 5 µL of cDNA template (diluted 1:10 in 0.1× TE buffer) and 15 µL of PCR reaction mix, containing 10 µL of TaqMan^®^ Fast Advanced Master Mix (2×), 1 µL of TaqMan^®^ Advanced miRNA Assay (20×) (see Table 5 for mature miRNA sequences), and 4 µL of RNase-free water. The thermal cycling protocol included an initial enzyme activation step at 95 °C for 20 s, followed by 40 amplification cycles of denaturation at 95 °C for 3 s and annealing/extension at 60 °C for 30 s.

The cycle threshold (Ct) for each miRNA was automatically determined using baseline and threshold settings in the 7500 Fast Real-Time PCR software (version 2.3). Relative expression levels were quantified using the comparative threshold cycle (2^−ΔΔCt^) method with miR-16 as endogenous reference control, a widely accepted approach for relative gene expression analysis in real-time PCR studies [48]. For statistical analysis, ΔCt values (miR of interest—miR-16) were compared across the three study groups: Control, MAP, and SAP. Fold-changes in expression were calculated as 2^−ΔΔCt^, using the mean ΔCt of the Control group as the calibrator.

### 4.6. Statistical Analysis

Statistical analysis and graphical representations were performed using MedCalc^®^ software version 14.8.1, except for the statistical analysis and graphical representations of ROC analysis, which were performed using R Studio software version 2025.05.1.

Data normality was assessed using both the Shapiro–Wilk and D’Agostino–Pearson tests. When the *p*-value was below 0.05, indicating deviation from normality, non-parametric tests were applied. Group differences were analyzed using the Mann–Whitney U test. Comparisons of gender, sex, and geographical distribution were analyzed by a Chi-square test. Comparisons of nonparametric variables between groups were analyzed by a Mann–Whitney U test, and between parametric variables by using a paired *t*-test. Correlations between miRNA levels and clinical variables were analyzed using Spearman’s rank correlation coefficient.

The diagnostic performance of selected miRNAs as potential biomarkers for differentiating AP cases from normal controls, as well as for discriminating between MAP and SAP, was further evaluated using receiver operating characteristic (ROC) analysis based on both ΔCt and 2^−ΔΔCt^ values. Diagnostic accuracy was quantified in terms of the area under the curve (AUC), sensitivity (Sn), and specificity (Sp) [49].

## 5. Conclusions

In conclusion, this study demonstrates that circulating miR-21, miR-122, miR-145, and miR-146a are significantly dysregulated in AP and correlate closely with disease severity. Specifically, miR-21 and miR-122 were upregulated, whereas miR-145 and miR-146a were downregulated, particularly in cases of SAP. Among the individual markers, miR-146a showed the best diagnostic performance; however, the combined multi-miRNA panel provided the highest accuracy in differentiating MAP from SAP.

Ultimately, these circulating miRNAs represent a robust, non-invasive adjunctive and complementary tool that, when integrated with established scoring systems, enhances early risk stratification. This combined approach allows for a more refined clinical management of AP, offering a potential advantage in the early identification of patients requiring intensive monitoring and personalized therapeutic interventions.

## Figures and Tables

**Table 1:** Demographic and clinical variables of the MAP patients, SAP patients, and the control group.

Variables	MAP (*n* = 23)	SAP (*n* = 27)	Control (*n* = 24)	*p* *^a^*	*p* *^b^*
Age (y), mean ± SD	56.78 ± 15.85	64.55 ± 16.92	55 ± 16.76	0.29	0.46
Sex (F/M), N	9/14, 23	10/17, 27	12/12, 24	0.21	0.98
Area (Rural/Urban), N	12/11, 23	8/19, 27	0/24, 24	0.83	<0.01
Balthazar (C/D/E), N	21/0/1, 22	1/5/21, 27	-	-	0.55
AMY (U/L), mean (min–max)	722.89 (34–3280)	1262.14 (120–3665)	-	-	<0.01
LPS (U/L), mean (min–max)	1698.33 (22.2–15,200)	1533.07 (115–4059)	-	-	0.18
Ca (mmol/L), mean (min–max)	1.05 (0.6–1.26)	1.02 (0.5–1.5)	-	-	0.29
Blood sugar (mmol/L), mean (min–max)	189.90 (63–1204)	221.03 (62–1204)	-	-	0.32
TG (mg/dL), mean (min–max)	421.55 (89–2821)	354.20 (101–2500)	-	-	0.58
Ranson	3.63 (1–7)	5.18 (2–9)	-	-	0.01
APACHE II	8 (0–27)	15.33 (1–34)	-	-	<0.01
CRP	12.58 (3.46–27)	26.41 (2.6–54.5)	-	-	<0.01
Leukocytosis (Y/N), N	14/9, 23	23/4, 27	-	-	0.29
ESR	44.35 (1–141)	55.42 (3.9–141)	-	-	0.44
Etiology					
Biliary (Y/N), N	8/15, 23	13/14, 27	-	-	0.24
Ethanol (Y/N), N	6/17, 23	7/20, 27	-	-	0.82
Metabolic (Y/N), N	2/21, 23	6/20, 26	-	-	0.97
Post interventional (Y/N), N	5/18, 23	2/25, 27	-	-	0.90
Dysfunctions					
Neurological (Y/N), N	20/3, 23	12/15, 27	-	-	0.93
Cardiovascular (Y/N), N	4/19, 23	14/13, 27	-	-	0.79
Respiratory (Y/N), N	4/19, 23	12/15, 27	-	-	0.64
Renal (Y/N), N	6/17, 23	13/14, 27	-	-	0.72
Fluidocoagulant (Y/N), N	7/16, 23	16/11, 27	-	-	0.95
Hematological (Y/N), N	6/17, 23	14/13, 27	-	-	0.91

*^a^* AP group versus control group. *^b^* MAP versus SAP.

**Table 2:** Correlations between miRNA levels in serum and clinical variables from the AP patient samples.

Variable	miR-21 (*r; p*)	miR-146a (*r; p*)	miR-145 (*r; p*)	miR-122 (*r; p*)
Sex	*r* = 0.15; *p* = 0.29	*r* = −0.07; *p* =0.58	*r* = 0.06; *p* =0.65	*r* = 0.20; *p* =0.15
Age	*r* = 0.07; *p* = 0.62	*r* = 0.002; *p* = 0.98	*r* = −0.14; *p* = 0.32	*r* = 0.20; *p* = 0.14
Geographical distribution	*r* = 0.10; *p* = 0.48	*r* = −0.14; *p* = 0.32	*r* = −0.29; *p* = 0.03	*r* = 0.24; *p* = 0.08
Balthazar	*r* = 0.38; *p* < 0.01	*r* = −0.26; *p* =0.06	*r* = −0.54; *p* < 0.01	*r* = 0.43; *p* < 0.01
AMY	*r* = 0.03; *p* = 0.79	*r* = −0.43; *p* < 0.01	*r* = −0.22; *p* = 0.11	*r* = 0.009; *p* = 0.95
LPS	*r* = −0.12; *p* = 0.39	*r* = −0.11; *p* = 0.41	*r* = 0.21; *p* = 0.14	*r* = −0.02; *p* = 0.88
Ca	*r* = −0.2; *p* = 0.04	*r* = −0.03; P = 0.81	*r* = 0.02; *p* = 0.88	*r* = −0.20; *p* = 0.18
Blood sugar	*r* = −0.14; *p* = 0.32	*r* = 0.26; *p* = 0.07	*r* = 0.15; *p* = 0.30	*r* = 0.24; *p* = 0.08
TG	*r* = −0.08; *p* = 0.66	*r* = −0.11; *p* = 0.54	*r* = −0.10; *p* = 0.57	*r* = −0.14; *p* = 0.44
Ranson	*r* = 0.27; *p* = 0.05	*r* = 0.15; *p* = 0.29	*r* = −0.20; *p* = 0.16	*r* = 0.40; *p* < 0.01
Apache II	*r* = 0.28; *p* = 0.04	*r* = 0.10; *p* = 0.47	*r* = −0.19; *p* = 0.18	*r* = 0.49; *p* < 0.01
CRP	*r* = 0.18; *p* = 0.32	*r* = −0.13; *p* =0.48	*r* = −0.29; *p* = 0.11	*r* = 0.25; *p* = 0.17
Leukocytosis	*r* = 0.30; *p* = 0.03	*r* = −0.24; *p* = 0.09	*r* = −0.23; *p* = 0.10	*r* = −0.04; *p* = 0.74
ESR	*r* = 0.24; *p* = 0.20	*r* = 0.18; *p* = 0.32	*r* = 0.03; *p* = 0.87	*r* = 0.34; *p* = 0.06
Etiology				
Biliary	*r* = 0.12; *p* = 0.38	*r* = 0.01; *p* = 0.90	*r* = 0.05; *p* = 0.72	*r* = 0.11; *p* = 0.42
Ethanol	*r* = −0.04; *p* = 0.73	*r* = 0.15; *p* = 0.27	*r* = 0.04; *p* = 0.75	*r* = 0.17; *p* = 0.21
Metabolic	*r* = −0.04; *p* = 0.78	*r* = −0.04; *p* = 0.76	*r* = −0.19; *p* = 0.17	*r* = 0.14; *p* = 0.30
Post interventional	*r* = −0.11; *p* = 0.44	*r* = −0.08; *p* = 0.55	*r* = −0.04; *p* = 0.77	*r* = −0.10; *p* = 0.44
Dysfunctions				
Neurological	*r* = 0.23; *p* = 0.10	*r* = 0.09; *p* = 0.51	*r* = −0.09; *p* = 0.51	*r* = 0.45; *p* < 0.01
Cardiovascular	*r* = 0.20; *p* = 0.15	*r* = 0.007; *p* = 0.95	*r* = −0.14; *p* = 0.32	*r* = 0.36; *p* < 0.01
Respiratory	*r* = 0.19; *p* = 0.17	*r* = 0.08; *p* = 0.55	*r* = −0.03; *p* = 0.78	*r* = 0.31; *p* = 0.02
Renal	*r* = 0.08; *p* = 0.56	*r* = 0.17; *p* = 0.23	*r* = −0.08; *p* = 0.55	*r* = 0.40; *p* < 0.01
Fluidocoagulant	*r* = 0.12; *p* = 0.37	*r* = −0.03; *p* = 0.83	*r* = −0.23; *p* = 0.09	*r* = 0.42; *p* < 0.01
Hematological	*r* = 0.11; *p* = 0.44	*r* = −0.02; *p* = 0.88	*r* = −0.16; *p* = 0.24	*r* = 0.40; *p* < 0.01

**Table 3:** Receiver operating characteristic curve analysis of miR-21, miR-146a, miR-145, and miR-122.

miRNA	AUROC (CI; *p* Value)	Sensitivity	Specificity	Cut-Off
MAP				
miR-21	0.618 (0.463–0.757; *p* = 0.16	39.13%	86.96%	0.26
miR-122	0.445 (0.401–0.701; *p* = 0.52)	95.65%	21.74%	0.17
miR-145	0.645 (0.490–0.780; *p* = 0.08)	60.87%	69.57%	0.30
miR-146a	0.832 (0.692–0.926; *p* < 0.001)	86.96%	69.57%	0.56
SAP				
miR-21	0.787 (0.654–0.0886; *p* < 0.001)	59.26%	100.00%	0.59
miR-122	0.703 (0.563–0.820; *p* = 0.004)	74.07%	66.67%	0.40
miR-145	0.916 (0.808–0.974; *p* < 0.001)	96.30%	74.07%	0.70
miR-146a	0.944 (0.845–0.988; *p* < 0.001)	88.89%	92.59%	0.81

**Table 4:** AUC, sensitivity, and specificity of the combined panel of four miRNAs (miR-21, miR-122, miR-145, and miR-146a) in distinguishing MAP and SAP patients.

Combinations	AUROC (CI; *p* Value)	Sensitivity	Specificity	Cut-Off
MAP	0.858 (0.724–0.943; *p* < 0.001)	86.96%	78.26%	0.65
SAP	0.993 (0.921–1.000; *p* < 0.001)	96.30%	100.00%	0.96

**Table 5:** The mature miRNAs sequence.

Nr. Crt.	miRNA	Mature miRNA Sequence
1.	hsa-miR-21-5p	UAGCUUAUCAGACUGAUGUUGA
2.	hsa-miR-122-5p	UGGAGUGUGACAAUGGUGUUUG
3.	hsa-miR-145-5p	GUCCAGUUUUCCCAGGAAUCCCU
4.	hsa-miR-146a-5p	UGAGAACUGAAUUCCAUGGGUU
5.	hsa-miR-16-5p	CCAAUAUUACUGUGCUGCUUUA

## Data Availability

The data presented in this study are available on request from the authors.

## Abbreviations

The following abbreviations are used in this manuscript:

AP	Acute pancreatitis
MAP	Mild acute pancreatitis
SAP	Severe acute pancreatitis
CT	Computed tomography
APACHE II	Acute Physiology and Chronic Health Evaluation
mi RNA	MicroRNA
ESR	Erythrocyte Sedimentation Rate
PCR	Polymerase chain reaction
ROC	Receiver operating characteristic
AUC	Area under the curve
ERCP	Endoscopic retrograde cholangiopancreatography
SIRS	Systemic inflammatory response syndrome
MODS	Multiple organ dysfunction syndrome
mRNA	Messenger RNA
RT	Room temperature
cDNA	Complementary DNA
RT	Reverse transcription
Ct	Cycle threshold
Sn	Sensitivity
Sp	specificity
CRP	C-reactive protein
